# Drink and be merry? The impact of intoxication and affective social cues on social drinkers’ emotional responses

**DOI:** 10.1093/alcalc/agaf046

**Published:** 2025-07-25

**Authors:** Rebecca L Monk, Adam W Qureshi, Byron L Zamboanga, Anna Tovmasyan, Olivia McLaughlin, Megan Bradford-Priest, Amber Butler, Derek Heim

**Affiliations:** Department of Psychology, Edge Hill University, St. Helens Road, Ormskirk, Lancashire L39 4QP, United Kingdom; Department of Psychology, Edge Hill University, St. Helens Road, Ormskirk, Lancashire L39 4QP, United Kingdom; Department of Psychological Science, 216 Memorial Hall, University of Arkansas Fayetteville, Arkansas, 72701, United States; School of Psychology and Clinical Language Sciences, Earley, Reading RG6 6ET, United Kingdom; Department of Psychology, Edge Hill University, St. Helens Road, Ormskirk, Lancashire L39 4QP, United Kingdom; Department of Psychology, Edge Hill University, St. Helens Road, Ormskirk, Lancashire L39 4QP, United Kingdom; Department of Psychology, Edge Hill University, St. Helens Road, Ormskirk, Lancashire L39 4QP, United Kingdom; Department of Psychology, Edge Hill University, St. Helens Road, Ormskirk, Lancashire L39 4QP, United Kingdom

**Keywords:** alcohol, intoxication, emotion, groups, social, context

## Abstract

**Background:**

While alcohol’s ability to impact affective states and lubricate social interactions is well documented, less research has considered this in crowd contexts.

**Method:**

Using a Social Emotion Paradigm, intoxicated (.8 g/kg) or sober (placebo) participants (*N* = 47, 49% female, *M*_age_ = 21.47) were presented with virtually modeled groups of characters displaying various affective states (happy, neutral, sad). Participants’ emotional responses to the stimuli were assessed via self-report (Study 1) and, one week later, objective measures of facial muscle movement (facial electromyography; Study 2).

**Results:**

‘Study 1’: Self-reported emotions largely mirrored the emotive displays, pointing to emotional contagion. No significant effect of intoxication was apparent. ‘Study 2’: Compared to those in the sober conditions, significantly more smiling occurred among intoxicated participants when viewing sad crowds.

**Conclusion:**

Discrepancies between objective and subjective measures of emotion were evident and intoxication may be associated with socially inappropriate affective responses to sad crowds. These findings have implications for understanding alcohol behaviors in the nighttime economy.

Long considered a social lubricant (Kirchner et al. 2006, Frings et al. 2008, Sayette et al. 2012), alcohol’s capacity to shape social interactions is well documented (e.g. Monahan and Lannutti 2000, Kirchner et al. 2006, de Visser et al. 2013, Fairburn et al. 2015, Zhou et al. 2018). Studies of individuals and small groups show that alcohol can enhance positive perceptions of others (Aan Het et al. 2008, Fairburn and Sayette 2014), impact mood (Monk et al. 2020), and influence responses to social stimuli (Dericco and Garlington 1977, Garlington and Dericco 1977, Collins et al. 1985, Corcoran and Segrist 1993). However, the extent to which alcohol intoxication might shape affective responses to crowd emotions has yet to be explored, despite alcohol consumption being a feature of many events involving large groups of people.

The link between emotions and intoxication has been extolled in both academic and lay circles (see Babor et al.’s 1983 particularly read-worthy fable of Saint Martin’s emotional behavior following alcohol consumption). Indeed, extensive research attests to alcohol’s ability to elevate positive affect (Sayette 2017) and impact the perception/recognition of certain emotions. For example, it has been found that intoxication enhances the recognition of happy (Dolder et al. 2017) contemptuous and disgusted facial expressions (Felisberti and Terry 2015) while impairing recognition of fear and sadness (Honan et al. 2018), reducing empathetic accuracy (Thiel et al. 2018) and social cognition (Baltariu et al. 2023), and, in some cases, resulting in inappropriate emotional response to faces (e.g. Francis et al. 2019). Indeed, it has been suggested that there may be a myopic narrowing of attention (Steele and Josephs 1990, Erthal et al. 2005) or a “hyperfocus” on emotional stimuli (Mocaiber et al. 2011) associated with intoxication, and these processes may play a role in behavioral responses such as aggression (see Attwood and Munafò 2014, c.f., Attwood et al. 2009). It has also been speculated that intoxication may impact attention to and processing of emotional faces owing to the effect of inebriation on interoceptive cues (Leganes-Fonteneau et al. 2021, c.f. Leganes-Fonteneau 2024). Consequently, both research and theory illustrate the link between alcohol and emotions and the ways in which various social influences can shape these associations (e.g. Monk and Heim 2014, Monk et al. 2015, Peacock et al. 2015, Austin et al. 2020, De Leon et al. 2020, Tovmasyan et al. 2022a, 2022b, 2023).

Noting calls for alcohol administration research to be more sensitive to contextual and social influences (Fairburn et al. 2015, Sayette 2017), exploring the interplay between social context, emotions and consumption may, however, be difficult in real-world environments. This is because there are numerous confounds in in vivo research (e.g. unknown levels of intoxication, unobserved social interactions). As such, laboratory assessments combining social-contextual cues and alcohol consumption have been useful to study alcohol-related beliefs and behaviors while cueing and measuring emotions. Many studies conducted on this topic have, however, exclusively relied on self-report (Sayette 2017), which is a limitation for a number of reasons: First, there are questions around people’s ability to accurately recall their alcohol consumption (Schwarz 1999, Monk et al. 2015), particularly while intoxicated (Walker and Hunter 1978), as well as questions as to whether people can reliably self-introspect and report on their affective states (Seager 2002). Second, the interactive and changing nature of emotion, intoxication and testing context (Tovmasyan et al. 2023) renders this a difficult task. Third, there are concerns that self-report measures are susceptible to demand characteristics (Nisbett and Wilson 1977) and may not be sensitive enough to detect or capture differences in emotional experiences (Fredrickson and Levenson 2011). Moving beyond self-report, work by Sayette et al. (2012) indicates that intoxication is associated with indicators of positive affect (e.g. smiling), while inebriation removed gender-based differences in the emotional contagion of smiles (observed in sober respondents) among social groups (Fairburn et al. 2015). This formative research has been important in highlighting the complex interplay between intoxication and social context shaping individuals’ (emotional) responses.

Despite prior research and theory highlighting the interplay between the social context, affect, and intoxication, the question of how crowds impact the emotions of others has not received attention in the alcohol field. This is even though alcohol consumption frequently occurs in environments where crowds are a prominent feature, such as in the nighttime economy (Levine et al. 2012), El Botellón (Pedrero-García 2018), sporting events (Neal and Fromme 2007, Leavens et al. 2019), and music festivals (Earl et al. 2004). Moreover, as people may decide to run away ‘with’ a crowd (e.g. to seek safety), or to run away ‘from’ an angry crowd (Carlsson et al. 2004, Alpers et al. 2009) based on how they interpret crowd emotions, a better understanding of how emotions spread from crowds to individuals under the influence of alcohol could have practical implications for the management of intoxicated crowds. To explore this issue, we draw on the research contributions from the crowd emotional contagion literature as our starting point.

By now it is known that individuals use others’ emotional cues, with research documenting that not only are people responsive to facial emotions (e.g. Hatfield et al. 2014a, 2014b, Richler and Gauthier 2014), but they also take affective cues from the bodily movements of others (Aviezer et al. 2012), their configuration (de Gelder 2006, Tamietto and de Gelder 2009, de Gelder et al. 2010) and gait (Schneider et al. 2014). While work in this field has been overly reliant on using “crowd” stimuli in the form of arrays of individuals faces or bodies (e.g. Lundqvist 1995, Lundqvist and Dimberg 1995, Wild et al. 2001), recent work has introduced a new paradigm which allows researchers to use more dynamically modeled crowds displaying both heterogeneous and homogonous emotions (see Qureshi et al. 2024). This research found that individuals paid more attention (higher % dwell times) to positive (vs sad) emotional crowd stimuli and that concordant happy and sad emotions were reported in observers of happy and sad crowds, respectively.

The current study builds on this literature by examining how intoxication impacts emotional contagion from crowds. It utilizes alcohol/placebo administration procedures and the Social Emotion Paradigm (Qureshi et al. 2024) where participants are exposed to videos of virtual crowds which have been modeled to reflect real life crowd movements and bodily expressions of emotions among interacting characters. Moving beyond a sole reliance on self-reported affect (Curtin and Lang 2007), it also uses facial electromyography (EMG) to provide a more unfiltered and implicit measure of affective response (see research in advertising, e.g. Hazlett and Hazlett 1999, Liao et al. 2015, as well as work with Facial Action Coding Systems in the study of alcohol, e.g. Sayette et al. 2012, Fairburn et al. 2015). However, it is worth cautioning that some research suggests that viewing emotional faces engages attention processes faster than when viewing emotional scenes, while facial mimicry responses (assessed via EMG), appear weaker during exposure to emotional faces versus scenes (Mavratzakis et al. 2016). We therefore use EMG in the knowledge that previous work has yielded different effects on emotional responses (see Mocaiber et al. 2011 for a review), and also note that the impact of intoxication on emotional recognition and affective response has produced similarly mixed results (Capito et al. 2017).

In short, across two studies, our aim is to examine the extent to which emotional contagion from crowds to individuals is impacted by alcohol intoxication. The first study (Study 1) examines self-reported emotional contagion while the second study (Study 2) assesses muscle movement (smiling and frowning) using facial EMG. In line with previous research (Qureshi et al. 2024) and the tenants of emotional contagion, we hypothesized greater self-reported happiness in response to happy social scenes, whilst greater sadness was expected in response to sad displays. Smiling was expected in response to happy group displays and frowning was anticipated in response to sad scenes (Seibt et al. 2015) (**Hypothesis 1-H1**). In light of the established effects of alcohol consumption on mood (Cooper et al. 1995, Sayette 2017, Monk et al. 2020) and its influence on emotion recognition (Dolder et al. 2017, Thiel et al. 2018), it was also postulated that intoxicated (relative to sober) participants would experience stronger emotional reactions in response to social stimuli (i.e. more happy/sad in response to happy/sad stimuli), when measured by both self-report and facial EMG (**Hypothesis 2-H2**). Finally, we hypothesized that alcohol’s myopic narrowing of attention would result in stronger affective responses from foreground (versus background) characters (**Hypothesis 3-H3**).

## Method

### Transparency and openness

All data, analysis information, and research materials are available at https://osf.io/4swjy/?view_only=ac7e9e74953f4b009088e12a42bdd0e0. We report how we determined our sample size, all data exclusions, all manipulations, and all measures in the study.

### Participants

Sixty participants (Nine participants were removed prior to the main analyses for failing to attend both studies or for technical issues with the facial EMG hardware in Study 2. A further four were removed as per the analytical strategy (z scores above 3.5 by condition × condition × group. This resulted in the final analytical sample of 47) who were predominantly White British (95%) were recruited from a UK University to take part in Study 1, with a total of 47 in the final sample for analyses (self-report; 24 men, *M*_age_ = 21.47/*SD* = 3.08), range 18–24 years) and all 47 returned one week later for Study 2 (facial EMG). Participants were randomly allocated into either alcohol or placebo conditions in both studies. Descriptive statistics for each study are shown in Table 1. No differences were shown between conditions for either study (all *P*’s > .05).

**Table 1:** Descriptive statistics.

	TLFB	AUDIT	M	F	Age
Placebo (*n* = 23)	22.87 (11.70)	11.91 (4.16)	10	13	21.7 (3.04)
Alcohol (*n* = 24)	28.83 (20.65)	12.13 (4.59)	14	10	21.25 (3.18)
TLFB: *t*(45) = 1.211, *P* = .23 (*d* = 16.88); AUDIT: *t*(45) = .17, *P* = .87 (*d* = 4.38); Gender: *Χ*^2^ (1, *N* = 47) = 1.04, *P* = .31					

Note. Time Line Follow Back (TLFB) and Alcohol Use Disorders Identification Test (AUDIT) scores.

Apriori power analyses using G*Power 3.1.9.2 (Faul et al. 2009) suggested that to achieve an observed power of .95 for main effects and interactions with an expected large effect size (Previous studies using a similar paradigm saw a η_p_^2^ value of .63 for the main effect of emotion), a minimum sample size of 22 was required for Study 1. The sample size for Study 2 was based on Study 1, and the inclusion criteria were identical across both studies. All participants reported that they consumed alcohol at least twice a week, did not suffer from any medical conditions, nor were they on medication that may react negatively to alcohol consumption. Participants were deemed unfit to take part if they had previously suffered from an alcohol use disorder or if their responses to the Alcohol Use Disorders Identification Test (AUDIT; Saunders et al. 1993) were above 19 (indicative of warranting further diagnostic evaluation). Similarly, the protocol stipulated that participants would be excluded from the study if they presented with breath alcohol contents above zero prior to testing commencement. No participants were excluded for any of these reasons in the current study.

### Procedure, materials, and equipment

All research was ethically approved by the University’s ethics panel prior to data collection and conducted in accordance with the World Medical Association’s Declaration of Helsinki and APA ethical standards. Participants responded to recruitment advertisements that asked for native English speakers who consume alcohol at least twice a week. The advertisements detailed medical exclusion criteria (e.g. history of alcohol use disorder) and indicated that participation would necessitate alcohol consumption (vodka, orange and tonic to a maximum of 200 milliliters of vodka, calculated based on their weight and gender), before watching a series of short videos displaying an array of social scenes. Prior to attending the study, participants completed a medical screening questionnaire to determine study suitability, along with the AUDIT (Saunders et al. 1993) (The Alcohol Use Disorders Identification Test (AUDIT; Saunders et al. 1993) was used to assess alcohol consumption and drinking behaviors. This is a 10-item questionnaire with a Likert response scale anchored between 0 (*Never*) and 4 (4 or more times). Responses to this questionnaire showed excellent internal consistency, Cronbach’s *a =* .82, with a mean of 6.36 (*SD* = 3.52)) and The Time Line Follow Back (An established measure which guides participants to record their drinking over two weeks, by asking them to recall and recount the number of drinks consumed on each day of the 2 weeks prior to assessment) (TLFB; Sobell and Sobell 1990) questionnaire.

All study sessions took place in the laboratories between 12 and 6 pm. Participants were told they should refrain from drinking for at least 12 hours and from eating for 3 hours before participation. All participants provided a breath alcohol level of .0 mg/l (Lion Alcometer 400, Lion Laboratories, Vale of Glamorgan, United Kingdom) before commencing each study session. Upon attending, participants were given an information sheet, followed by an alcohol disclaimer, an informed consent form to complete, and, if they were women, a pregnancy disclaimer form to sign. Participants were then randomly assigned into an alcohol condition or a placebo condition and tested individually (this same random allocation process occurred for those who returned for Study 2).

### Alcohol administration

Participants were administered a volume of alcohol based on their weight and gender. Participants were given a combination of vodka (37.5% in strength), pure orange juice, and Indian tonic water as part of the study. Within the alcohol condition, alcohol was distributed in measurements of 0.8 g/kg based on the weight and gender of each participant (Rose and Grunsell 2008). The maximum amount of alcohol that participants could receive was 200 milliliters of vodka per session. Those participants assigned to the placebo condition received only orange juice and tonic water. A small spray containing trace amounts of Vodka was applied to the rim of the glasses to give the impression of alcoholic content (>2 ml), a procedure akin to Stafford et al. (2020). All solutions were prepared in a separate room using a measuring jug before they were poured into three tumbler glasses and presented to participants for consumption.

All participants were required to consume a strong mint to mask the taste of the alcohol (Hopthrow et al. 2007) and then were given 10 minutes to consume the total volume of the presented mixtures, followed by a 20-minute rest/absorption period prior to completing any other experimental tasks. This ensured that those in the alcohol condition were at the peak level of intoxication (see Rose and Duka 2006). Participants were breathalyzed again after the consumption and absorption periods. They were able to continue with the study if their BAC registered over 0.14 mg/l at this stage (there were no exclusions based on this criterion and all participants finished their drinks without reporting nausea).

Participants then completed the Social Emotion Paradigm (outlined below). Upon completion, participants were thanked for their time and offered the opportunity to take part in Study 2. All participants remained blind as to their study condition(s) until the cessation of their involvement with the research, which could happen at two time points: At the end of Study 1 (if participants declined the invitation to attend a second study, 1 week later) or at the end of Study 2 (if they signed up to return 1 week later (Participants who indicated their intention to return but did not subsequently were emailed the comparable debriefing information regarding alcohol and placebo testing conditions)). Participants who returned one week later underwent the same screening and alcohol administration procedures before Study 2 commenced.

For both studies, participants were allowed to leave once their BAC score was below 0.14 mg/l or if they signed a disclaimer stating that they were voluntarily leaving before this time, were not experiencing any negative effects from having consumed alcohol and would not drive, ride a bike, operate machinery, or exercise for at least 4–5 hours.

### The social emotion paradigm

This paradigm, developed by Qureshi et al. (2024), uses dynamic social stimuli depicting the interactions of individual characters in social groups, moving away from static facial stimuli (in arrays) such as those used in more traditional examinations of ‘crowd’ influence (e.g. the face in the crowd paradigm; Shasteen et al. 2014). The individuals displayed within these social stimuli are based on corpora of human body emotional behavior (Carnegie-Mellon Graphics Lab Motion Capture Database; UCLIC Affective Posture and Body Motion Database, Kleinsmith et al. 2006) and principles of emotional contagion (Carretero et al. 2014).

Previous research (Carretero et al. 2014, Qureshi et al. 2024) and pilot testing suggest that the bodily emotions represented in these stimuli are accurately recognized by observers and can impact self-reported affect in observers (For both individual (*F* (2, 60) = 201.13, *P* < .001, η_p_^2^ = .87) and crowds displays (*F* (2, 60) = 195.07, *P* < .001, η_p_^2^ = .87), there were main effects of body movement type, with happy body movements rated as significantly more positive than neutral and sad body movements, with sad movement rated as significantly more negative than neutral body movements (for both sets of stimuli; *P*’s < .001)). Characters were androgynous mannequins with no facial expressions or any other details that may have influenced participants (e.g. clothes or fingers), other than a small nose to make it easier to infer the direction of characters’ heads (Carretero et al. 2014).

The scenes displayed a group of three foreground agents that were interacting, with a larger crowd of 200 agents in the background (half interacting in groups while the rest walked through the crowd). The stimuli were set in a neutral environment (in front of KTH Royal Institute of Technology, Sweden).

**Figure 1:**
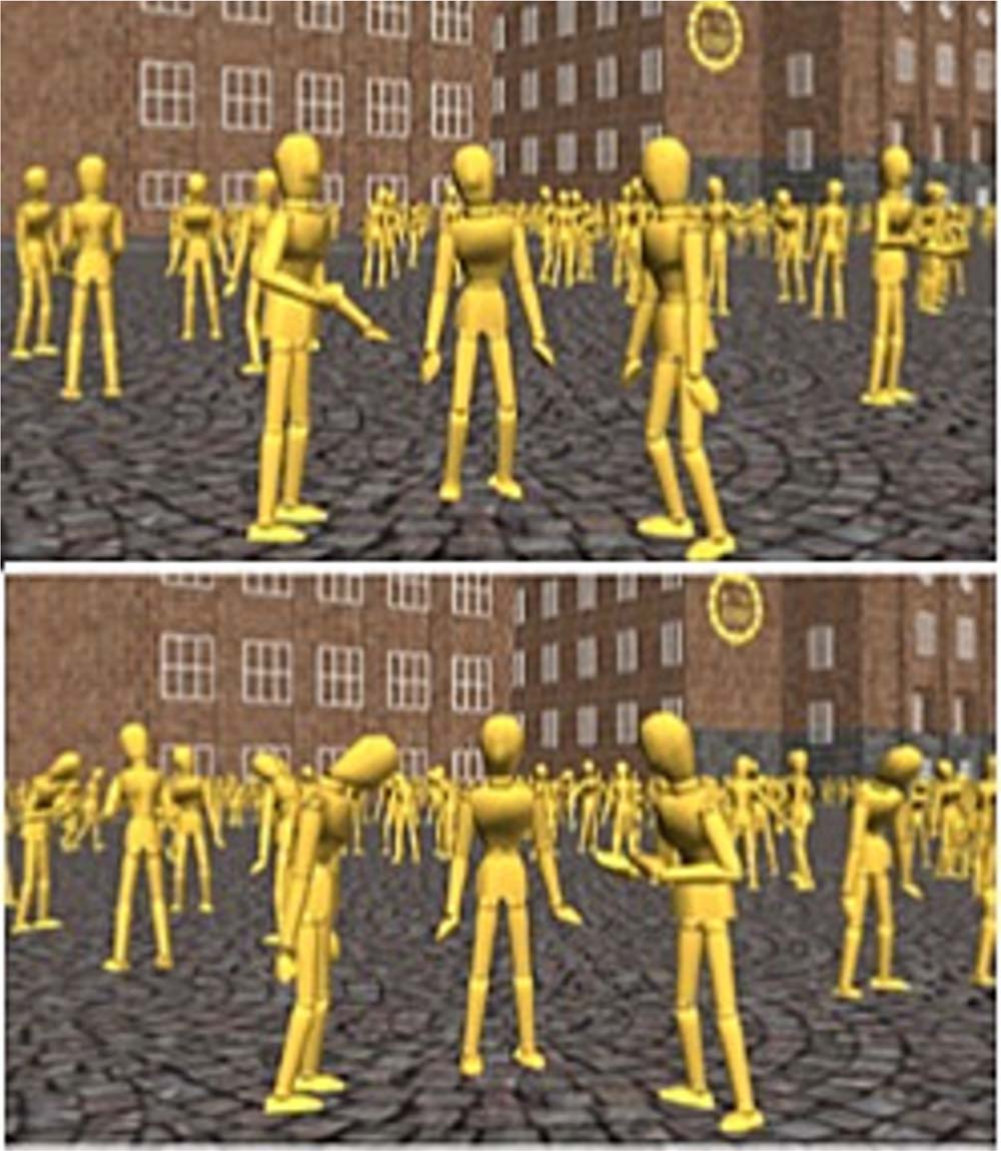
Examples of stimuli containing groups of characters exhibiting neutral (top) and sad (bottom) emotions. Three foreground characters engage in a conversation, surrounded by a background crowd of static groups and walkers

The agents in the stimuli displayed neutral, happy, and sad emotions. Examples of the stimuli can be seen in Fig. 1 and an exemplar video is available in the additional materials (Video 1_examplar stimuli.mp4). While crowds often behave in unison, this is not always the case, and it is important to understand the extent to which crowds that are in or out of sync may impact how individuals respond to them (see Elias et al. 2017). As such, foreground/background emotions were varied to afford six different combinations, following the protocol of Qureshi et al. (2024), which tests crowd emotional variability whilst limiting the time demands placed on participants: foreground characters’ emotion (FG*e*) = neutral or sad; background emotion (BG*e*) = happy, neutral or sad. There were six iterations of each combination of emotions, meaning that in total, each participant viewed 36 emotional displays in random order.

### Study 1: self-report

Participants were asked to watch the footage on a computer screen and, in between each video clip (lasting approximately 15 s each), they were given the opportunity to rate how they felt on a scale of 0–100 using a visual analog scale (0 = sad, 50 = neutral, 100 = happy), a response scale mirroring works on alcohol-related happiness (Peacock et al. 2015, Austin et al. 2020). Participants were debriefed, thanked for their time and remunerated, and offered the opportunity to take part in Study 2.

### Study 2: facial EMG

According to self-perception theory (Bem 1972), people infer their own attitudes and emotions by observing their behavior. Similarly applied to smiling, studies of embodied cognition (Strack et al. 1988) and the facial feedback hypothesis (Buck 1980) suggest that facial responses may inform self-reported emotions. To avoid possible conflation between self-report and facial responses, participants therefore took part in Study 2 one week following participation in Study 1. The procedure was identical to that of Study 1, the only exception being that within the Social Emotion Paradigm, participants were required only to watch the footage while facial EMG was used to record affective response. All other facets of the paradigm were the same and stimuli were randomized to help minimize order effects.

Facial muscle movement was measured using a Biopac MP36 unit, which was linked to a laptop (SPECS) running Biopac Pro and a desktop PC (SPECS) running the scenarios on E-Prime 2.0. Electrodes were plugged into the electrode adapter of the EMG; CH1 and CH2 on the MP36 Biopac unit. The MP36 data acquisition unit was then turned on, and BSL PRO software launched on the laptop. Electrodes were affixed to the face of each participant using conductive gel, adhesive collars, and a pipette. Abrasive pads were used, if required, for makeup removal.

The electrode from the CH1 lead was placed onto the corrugator supercilii muscle locations on the left side of the face; the negative lead was located slightly above the eyebrow, the positive lead 1 cm away between the negative lead and the nose, and the ground lead on the forehead. The CH2 lead was then placed on the zygomaticus major muscle, also on the left side of the face; the negative lead was placed diagonally between the mouth and the cheekbone, and the positive lead was placed 1 cm above the negative lead. The scenarios were synchronized with the MP36 output such that pulses were sent from the experiment using the digital channel at the start and end of each scenario, allowing specific scenarios to be linked to the relevant areas of muscle movements’ waveforms. This allowed the maximum amplitude of facial muscle movement (smile; zygomaticus major, frown; corrugator supercilii) to be calculated and exported for each video scenario.

The wires from these electrodes were tucked behind the participant’s ear and clipped to the back of their chair to avoid interference in the next part of the study. Muscle movements were checked against the display to ensure that movements were recorded before the study began. After viewing all the clips, the study was completed, the electrodes were removed by the researcher, and participants were thanked and debriefed.

### Analytic strategy

Prior to the main analyses, data were screened by condition (foreground character emotion, background character emotion) and group (consumption condition), with standardized values calculated for each combination. Participants with scores z-scores above 3.5 were removed from the analyses, resulting in a final sample size of 47.

2 × 3 × 2 mixed ANOVAS were conducted on self-reported EC (Study 1) and mean maximum muscle movement of the corrugator supercilii (frown), and zygomaticus major (smile) muscles (Study 2 (A 50 Hz High pass finite impulse response (FIR) filter was used post-acquisition)). The within-participants variables were foreground character emotion (FG*e*: neutral × sad) and background emotion (BG*e*: happy × neutral × sad), and the between-participants variable was consumption condition (alcohol or placebo). Any interactions were analyzed further using simple main effects with Bonferroni corrections to control for multiple comparisons (Supplementary analyses (carried out upon peer review request) revealed that neither the gender of the participants nor their level of alcohol-related harm (AUDIT) impacted the results and so these are not reported).

## Results

### Study 1

There was a main effect of foreground emotion (*F*(1, 45) = 46.47, *P* < .001, η_p_^2^ = .51), with more negative self-reported emotion when foreground characters were sad. There was also a main effect of background emotion (*F*(2, 90) = 79.16, *P* < .001, η_p_^2^ = .64), with more positive self-reported emotion when the background crowd were happy (*P*’s < .001), though there was no difference shown in response to neutral and sad crowds (*P* = .052). There was no main effect of consumption condition (*F*(1, 45) = .80, *P* = .38, η_p_^2^ = .02). There was an interaction between foreground and background emotion (*F*(2, 90) = 3.80, *P* = .026, η_p_^2^ = .08), but no other interactions.

**Figure 2:**
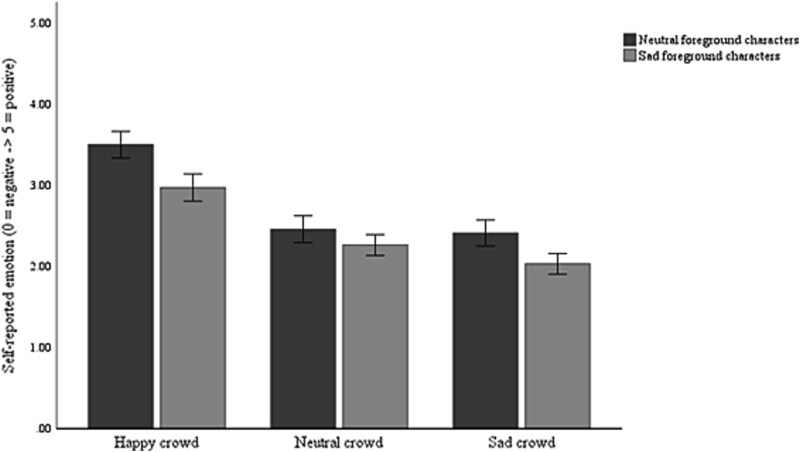
Self-reported emotional contagion by foreground characters and background crowd. Note. Error bars represent 95% confidence intervals

The interaction between foreground and background emotion was explored further using simple main effects. These show that while self-reported emotion was more negative when foreground characters were sad (relative to neutral) when the background crowd was happy or sad (*P*’s < .001), there was no difference when the background crowd was neutral (*P* = .09). When the foreground characters were sad, self-reported emotion followed the background crowd emotion, with the most positive ratings when they were happy, followed by neutral then sad (all *P*’s < .001). A similar pattern was shown when the foreground characters were neutral, though there was no difference in response to neutral and sad background crowds (*P =* 1.00; Fig. 2).

### Study 2

#### Movement of corrugator supercilii (frown)

There were no main effects of foreground emotion (*F*(1, 45) = .092, *P* = .76, η_p_^2^ = .00), background emotion (*F*(2, 90) = 1.48, *P* = .23, η_p_^2^ = .03) or consumption condition (*F*(1, 45) = 1.02, *P* = .32, η_p_^2^ = .02). There were also no interactions.

#### Movement of zygomaticus major (smile)

**Figure 3:**
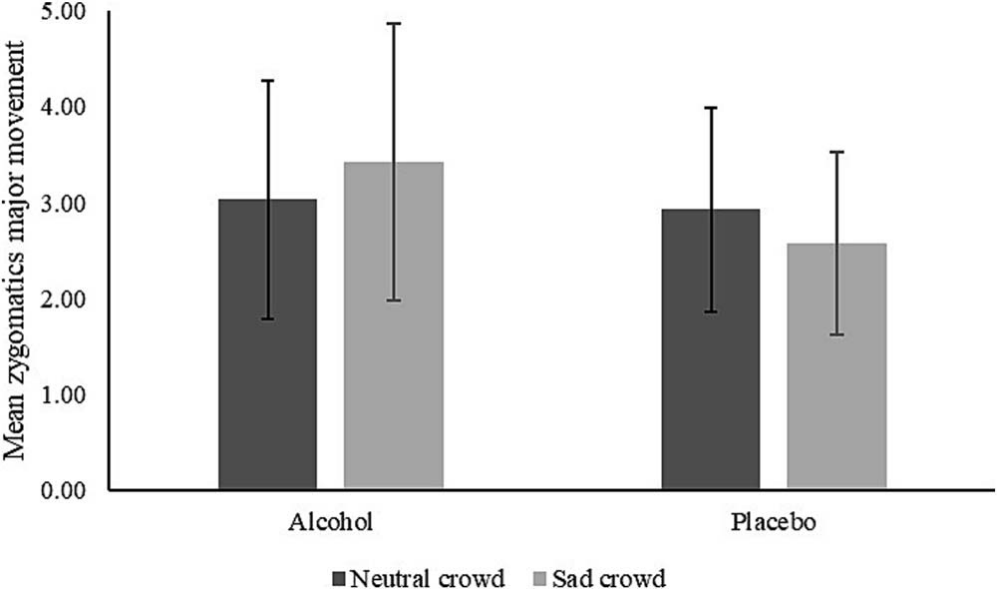
Interaction between foreground emotion and consumption condition on movement of zygomaticus major (smile)

There were no main effects of foreground emotion (*F*(1, 45) = .001, *P* = .98, η_p_^2^ = .00), background emotion (*F* (2, 90) = .54, *P* = .58, η_p_^2^ = .01) or consumption condition (*F*(1, 45) = .28, *P* = .60, η_p_^2^ = .01). There was an interaction between foreground emotion and consumption condition (*F*(1, 45) = .7.10, *P* = .01, η_p_^2^ = .14), but no other interactions (see Fig. 3). The interaction was explored further using simple main effects.

There were no differences in smiling between the consumption conditions in response to either neutral or sad foreground characters (*P*’s > .35). While there were also no differences in muscle movement in response to neutral and sad foreground characters for either consumption condition (placebo: *P* = .07, alcohol: *P* = .06), the pattern differed between these groups. Specifically, those in the placebo group smiled less when the foreground characters were sad (versus neutral), whereas those in the intoxicated group smiled more when the foreground characters were sad (versus neutral).

## Discussion

The current research examined the emotional impact of exposure to crowds during intoxication, and findings suggest that there may be a potentially complex interplay between alcohol consumption and emotional contagion from crowds, warranting further investigation. Using realistically modeled stimuli and building on prior research on the effect of emotion and intoxication in small groups (Sayette et al. 2012, Fairburn et al. 2015), our findings support the notion that social context is important in the study of alcohol consumption and affect (Sayette 2017). In accordance with research observations using individual emotional stimuli (e.g. Wild et al. 2001), findings indicate that emotions may also be “caught” from crowds, and viewing scenes displaying sadness elicited more frowns and fewer smiles in participants (**H1**). Self-reported emotion was also more negative when foreground characters were sad (relative to neutral) when the background crowd was happy or sad, which may provide some indication of a negativity bias (Ito et al. 1998, Baumeister et al. 2001, Smith et al. 2003, though see Qureshi et al. 2024) whereby more attention may be drawn to sad people, with a subsequent impact on the observers’ emotion (**H1**).

There was no evidence to support the hypothesis that a myopic narrowing of attention (Steele and Josephs 1990, Erthal et al. 2005) would result in stronger (contagious) affective responses from foreground characters when observed by intoxicated versus sober participants (**H3**). Our findings also do not support our hypothesis that intoxicated (versus sober) participants would experience stronger emotional responses in response to social stimuli when measured by both self-report and facial EMG (**H2**). Intoxication, as such, had no apparent impact on self-reported affect (positive or negative). However, rather than elevating contagious smiling in response to happy social scenes, there was evidence that inebriated participants smiled more at sad social stimuli than did their sober counterparts. As intoxicated participants’ self-reported emotions in the direction of the affect they observed, this finding does not appear to be driven by a lack of explicit emotion recognition, seen in some previous works (e.g. Attwood and Munafò 2014, Walter et al. 2011, Honan et al. 2018). Rather, this may indicate that alcohol can trigger socially inappropriate facial responses, as in the current study, intoxicated participants appeared more likely to smile at sad scenes than their sober counterparts. Given that social display rules provide people with a set of norms for responding in social contexts, it may therefore be the case that intoxication counteracts the inhibitory effect of social display rules (see Capito et al. 2017 for review). On the other hand, inebriation may ameliorate the perceived need to comply with social display rules (ibid); in this context, to refrain from smiling—or to frown—when others appear sad. Future research that builds on our studies is clearly required to investigate systematically such possibilities. Nevertheless, the present findings suggest a potential difference in the effect of alcohol on explicit (self-report) versus implicit (facial EMG) emotional responses and represent a novel contribution to our understanding of emotional contagion from crowds in alcogenic environments. These findings may have implications for future research with respect to the methods by which social group contagion effects are studied. They also have potential to impact our understanding of aggressive behaviors in the nighttime economy, suggesting that intoxicated people may not be able to discern or intuit negative affective cues in a crowd, leading to potentially inappropriate social responses. Recent research (Qureshi et al. 2024) indicates that emotional contagion from crowds may be more distinct than that experienced from observing individuals. Future research is therefore needed to examine if this also holds in the context of intoxication, expanding further our theoretical understanding in this domain.

### Limitations and future directions

First, the present research did not control for participants’ baseline emotions (which could impact attention, e.g. Lodder et al. 2016), and we cannot rule out individual differences in spontaneous mimicry (Laird et al. 1994) or the propensity to “catch” others’ emotions (Doherty 1997). It should also be acknowledged that participants in this research conducted the entirety of the study in isolation and were passive observers, meaning that the current study may not be entirely replicated to affective response ‘within’ real-life social interactions during intoxication. Future research should also seek to replicate the current foundational findings with greater power to detect smaller effect sizes, particularly in respect to the interactions, and implement a balanced placebo design (Rohsenow and Marlatt 1981) to further disentangle the pharmacological from anticipatory effects of alcohol. Further, given that neural response to negative emotional stimuli has been implicated in the transition to hazardous alcohol consumption (Kirk-Provencher et al. 2024), forthcoming explorations should also examine whether the current findings are replicated in clinical populations.

The nature of the current study design meant that participants were tested twice, and the order of participation in the two studies was not randomized. Therefore, we cannot discount the possibility of order or practice effects. Additionally, while the established association between emotional displays and self-reported emotions (Olszanowski et al. 2020) means facial movements are a useful tool for assessing affect, assessing smiling/frowning may not capture the full extent of affective responses. Objective tools such as the assessment of endocrine responses (e.g. cortisol levels; Schlotz 2011) or less invasive assessments of facial movements (e.g. Facial Action Coding System, Sayette et al. 2012, see Clark et al. 2020) may also offer further promise in this area of research. Finally, future research could develop the social stimuli used in this study to include positive foreground characters, and to increase ecological validity by including wider contextual information (see Nohlen et al. 2016), a greater array of emotions (e.g. anger or fear; Attwood and Munafò 2014), and emotive facial cues.

### Conclusion

Discrepancies observed in the current study between objective and subjective measures of affective responses require further investigation to better understand the effect of intoxication during social encounters in intoxigenic contexts and spaces. Notwithstanding the need for future work, findings of this work suggest alcohol intoxication may be associated with socially inappropriate affective responses to sad crowds, which may suggest that contagious negative affect from observing sad others is ameliorated by intoxication. As we seek to develop interventions that are sensitive to the various settings in which alcohol behaviors occur, particularly those pertaining to crowds, future explorations of the complex interplay between alcohol and social and affective contexts appear warranted.

## Data Availability

All data, analysis information, and research materials are available at https://osf.io/4swjy/?view_only=ac7e9e74953f4b009088e12a42bdd0e0.
